# Toll-like receptor-5 agonist Entolimod broadens the therapeutic window of 5-fluorouracil by reducing its toxicity to normal tissues in mice

**DOI:** 10.18632/oncotarget.1773

**Published:** 2014-02-23

**Authors:** Bojidar M. Kojouharov, Craig M. Brackett, Jean M. Veith, Christopher P. Johnson, Ilya I. Gitlin, Ilia A. Toshkov, Anatoli S. Gleiberman, Andrei V. Gudkov, Lyudmila G. Burdelya

**Affiliations:** ^1^ Department of Cell Stress Biology, Roswell Park Cancer Institute, Buffalo, NY; ^2^ Buffalo BioLabs, LLC, Buffalo, NY; ^3^ Cleveland BioLabs, Inc., Buffalo, NY

**Keywords:** TLR5, flagellin, chemotherapy, hematopoietic, gastrointestinal toxicity, tumor

## Abstract

Myelosuppression and gastrointestinal damage are common side effects of cancer treatment limiting efficacy of DNA-damaging chemotherapeutic drugs. The Toll-like receptor 5 (TLR5) agonist Entolimod has demonstrated efficacy in mitigating damage to hematopoietic and gastrointestinal tissues caused by radiation. Here, using 5-Fluorouracil (5-FU) treated mice as a model of chemotherapy-induced side effects, we demonstrated significant reduction in the severity of 5-FU-induced morbidity and increased survival accompanied by the improved integrity of intestinal tissue and stimulated the restoration of hematopoiesis. Entolimod-stimulated IL-6 production was essential for Entolimod's ability to rescue mice from death caused by doses of 5-FU associated with hematopoietic failure. In contrast, IL-6 induction was not necessary for protection and restoration of drug-damaged gastrointestinal tissue by Entolimod. In a syngeneic mouse CT26 colon adenocarcinoma model, Entolimod reduced the systemic toxicity of 5-FU, but did not reduce its antitumor efficacy indicating that the protective effect of Entolimod was selective for normal, non-tumor, tissues. These results suggest that Entolimod has clinical potential to broaden the therapeutic window of genotoxic anticancer drugs by reducing their associated hematopoietic and gastrointestinal toxicities.

## INTRODUCTION

Severe adverse side effects continue to be a major challenge of use of many conventional anticancer drugs. These include gastrointestinal (GI) damage with such symptoms as diarrhea, vomiting, GI mucositis and body weight loss, and hematopoietic (HP) damage causing leukopenia, thrombocytopenia, myelosuppression leading to immunosuppression and uncontrolled bacteremia [1-4]. These toxicities frequently prevent administration of sufficiently effective drug doses and can severely affect quality of life or even be fatal [4-6].

Entolimod™ (previously called CBLB502) is a derivative of bacterial flagellin that functions as an agonist of Toll-like receptor 5 (TLR5) and has demonstrated tissue protective effects against multiple types of insults. Entolimod treatment rescued lethally irradiated mice and non-human primates from HP, GI and cutaneous acute radiation syndromes by preventing radiation-induced loss of HP stem cells and early progenitors in the bone marrow, rescuing proliferating stem cells in crypts of the small intestine, and reducing the severity of dermatitis and mucositis [7, 8]. Entolimod also demonstrated tissue protective efficacy in mouse models of renal ischemia-reperfusion injury [9] and Fas-mediated hepatotoxicity [10], indicating that its protective effects are not restricted to radiation-induced damage. Importantly, Entolimod does not protect tumors from radiation therapy, but rather suppresses tumor growth through stimulation of antitumor immune responses [8, 10-12]. TLR5 agonistic agents like Entolimod have a notable advantage over other TLR agonists that have been considered for clinical use (TLR3, 4 and 7 [13]) in that the specific profile of cytokines induced following TLR5 stimulation does not include those that can lead to a harmful uncontrolled “cytokine storm”, such as IL-1 and TNF [13, 14].

Entolimod's mechanism of action involves TLR5-dependent NF-B- (and STAT3-) mediated induction of multiple factors that suppress apoptosis, scavenge reactive oxygen species (ROS), and induce tissue regeneration. Given the similarity in toxicities induced in normal GI and HP tissues by radiation and DNA-damaging chemotherapeutic drugs and the potent efficacy of these Entolimod-induced mechanisms in reducing radiation damage, we expected that Entolimod might demonstrate similar protective effects in the context of genotoxic chemotherapy. Therefore, in this study, we focused on 5-Fluorouracil (5-FU), a DNA-damaging anticancer drug commonly used alone or in combination with other chemotherapeutics for treatment of a number of different types of cancer including colon, stomach, head and neck, breast, etc. [15-21]. High-dose continuous treatment with 5-FU causes DNA damage in proliferating cells through inhibition of thymidylate synthase, a key component of DNA synthesis and repair pathways. In addition to tumors, HP and GI cells demonstrate 5-FU sensitivity [22, 23]. Hematological toxicity of 5-FU involves p53-mediated induction of ribosomal stress, which blocks translation [24, 25]. In addition, 5-FU stimulates inflammatory responses that cause additional damage through production of reactive oxygen species (ROS) and secretion of IL-1 and TNF [3] which may be mediators of a p53-independent mechanism of 5-FU toxicity in the small and large intestine [22]. Inhibition of IL-1 activity in small intestinal tissue was shown to prevent 5-FU-induced GI injury [26]. IL-6 is another cytokine found to be associated with toxic effects of chemotherapeutic drugs [3]; however, it has also been reported to protect intestinal tissues from toxic doses of 5-FU [27].

In this study, we demonstrated significant protective effects of Entolimod on the toxicity of 5-FU in two mouse strains, BALB/c and C57BL/6. IL-6 was defined as an essential component of the mechanism by which Entolimod protects mice against an HP but not GI damage. Importantly, the protective effect of Entolimod against 5-FU toxicity was selective to normal tissues as demonstrated in the mouse model of colon cancer.

## RESULTS

### Effect of Entolimod on 5-FU-induced mortality in mice

To determine whether Entolimod is capable of protecting normal tissues from the toxicity of 5-FU, we used two mouse strains, BALB/c and C57BL/6, which differ in their sensitivity to 5-FU. Mice were administered 5-FU alone or in combination with subsequent Entolimod treatment using different doses and schedules. Toxicity was assessed by monitoring body weight loss and mortality.

**Figure 1:**
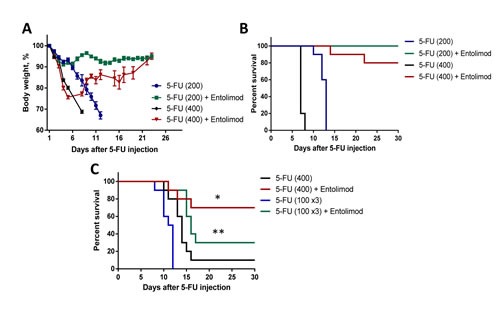
Effect of Entolimod on 5-FU-induced mortality in mice Body weight (A) and survival (B) of BALB/c mice treated with 5-FU (200 mg/kg or 400 mg/kg given in two equal fractions 6 h apart) alone or with subsequent injection of Entolimod (1 μg/mouse) 24 and 48 h after the last 5-FU dose (10 animals/group). Body weight is shown as a percentage of starting weight; mean ± SEM. C. Survival of C57BL/6 mice injected with a single dose of 400 mg/kg 5-FU or 3 daily doses of 100 mg/kg 5-FU with or without Entolimod (1 μg/mouse) injected 24 and 48 h after the last 5-FU dose; n=10 mice/group. The differences in mortality kinetics between corresponding 5-FU and 5-FU+Entolimod groups were statistically significant: (*) p<0.001; (**) p<0.03 by Log-rank test for 30-day survival.

In BALB/c mice, we found that injection of 100 mg/kg 5-FU induced only transient body weight loss without mortality, but that a single injection of 200 mg/kg 5-FU caused severe weight loss and 80-100% mortality within 2 weeks after 5-FU injection (Fig. 1A and B). The kinetics of body weight loss showed an initial drop in mean body weight during the first 3-5 days after injection, partial improvement by day 7-8, and then a second drop with no recovery. A higher dose of 5-FU (400 mg/kg) caused more rapid single-phasic body weight loss and death of 100% of animals within 7 days. At both tested doses of 5-FU, administration of Entolimod (two s.c. injections given 24 and 48 h after 5-FU) significantly reduced body weight loss and prevented mouse mortality. With 200 mg/kg 5-FU + Entolimod, mean body weight loss was minimal (<10%) and all mice survived to the end of the 30 day observation period. With 400 mg/kg 5-FU + Entolimod, the initial 5-FU-induced drop in mean body weight was observed, but this was reversed at Day 5, and on Day 30, mean body weight was restored to normal and 80% of animals were alive. Improved resistance of BALB/c mice to 5-FU-induced toxicity was also observed when Entolimod was applied 24 h (single injection), or 1, 48 and 96 h (three injections) after 200 mg/kg 5-FU as evidenced by substantially reduced weight loss and an increase in 30-day survival to 90% (versus 0% in the group given 5-FU alone) (Supplementary Fig. S1). Under the same conditions, two injections of Entolimod given 1 h and 24 h post-5FU prevented weight loss and mortality in only 3/10 mice, a statistically non-significant benefit.

C57BL/6 mice were more resistant to 5-FU toxicity than BALB/c mice. Administration of 200 mg/kg 5-FU to C57BL/6 mice was not lethal, but caused transient body weight loss (only about 10-15%) with complete recovery by Day 10 (data not shown). However, 400 mg/kg 5-FU, which caused early death of BALB/c mice by day 7, was lethal for majority of C57BL/6 mice by day 15 (Fig. 1C). Toxicity with similar kinetics was observed after three injections of 100 mg/kg 5-FU given 24 h apart, with severe weight loss and death occurring about 2 weeks post-treatment (Fig. 1C, Supplementary Fig. S2A-B). With both toxic regimens of 5-FU treatment, injection of Entolimod (1 μg/mouse) 24 and 48 h after injection of the last fraction of 5-FU resulted in significant reduction of body weight loss and improved survival. With addition of Entolimod to 400 mg/kg 5-FU treatment, 30-day survival was increased from 10% to 70% (p=0.0079, Fig. 1C). Similarly, when Entolimod was given to mice treated with 3x100 mg/kg 5-FU, mortality was significantly delayed. Mean survival time was increased from 11.5 days to 14 days (p= 0.0002) and 30-day survival was increased from 0% to 30%. The ability of Entolimod to reduce 5-FU-associated mortality was confirmed to be TLR5-specific since the effect was not observed in similarly treated TLR5 knockout mice (Supplementary Fig. S2C).

### Effect of Entolimod on 5-FU-induced hematopoietic damage

**Figure 2:**
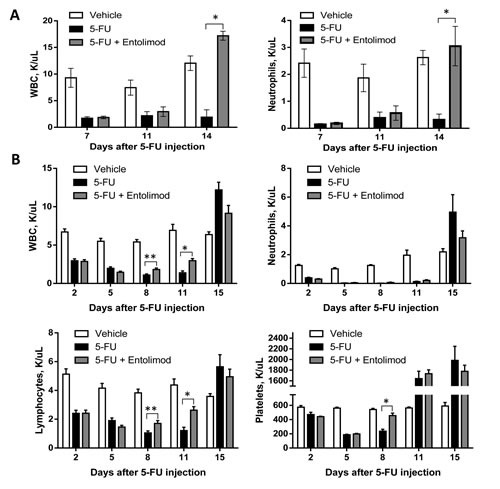
Protection and restoration of hematopoiesis Complete blood cell analysis was performed using blood samples from mice treated with vehicle (n=5), 5-FU alone (n=10) or 5-FU+Entolimod (n=10). 5-FU was injected i.p. at a dose of 200 mg/kg (A) or 100 mg/kg (B) followed by three s.c. Entolimod (1 μg/mouse) injections 1, 48 and 96 h post-5-FU. Mean concentrations (K/μl peripheral blood) of white blood cells (WBC), neutrophils, lymphocytes and platelets ± SEM are shown for the indicated days after 5-FU injection. The differences between 5-FU and 5-FU+Entolimod groups were statistically significant: (*) p<0.001; (**) p<0.05 by two-tailed unpaired t-test.

Since 5-FU-induced mortality can be prevented by Entolimod and is known to involve HP damage and Entolimod was previously shown to ameliorate radiation-induced HP damage, we next evaluated whether Entolimod specifically reduces HP damage caused by 5-FU. As shown in Figure 2, treatment of BALB/c mice with 200 or 100 mg/kg 5-FU resulted in rapid elimination of practically all types of blood cells, including neutrophils, lymphocytes and platelets, correlating with lethality observed within 11-14 days after 5-FU administration. Entolimod treatment after 200 mg/kg 5-FU did not significantly ameliorate 5-FU-induced depletion of blood cell populations as observed on Days 7 and 11 post-5-FU (Fig. 2A). However, in contrast to mice treated with 200 mg/kg 5-FU alone, mice treated with Entolimod after 200 mg/kg 5-FU administration showed gradual recovery of hematopoiesis leading to complete restoration of normal WBC and neutrophil levels by Day 14 and improved mouse survival. With sub-lethal doses of 100 mg/kg and 150 mg/kg 5-FU (Fig. 2B and Supplementary Fig. S3, respectively), mice treated with Entolimod after 5-FU consistently displayed more rapid recovery of peripheral blood cell populations than mice given 5-FU alone. In some cases in response to 5-FU with or without Entolimod, surviving mice displayed temporary overcompensation for the loss of HP cells: numbers of lymphocytes, neutrophils and platelets were higher in treated mice than in intact controls at the day 14-15 time-points (Fig. 2B), but normalized by day 21 (data not shown).

**Figure 3:**
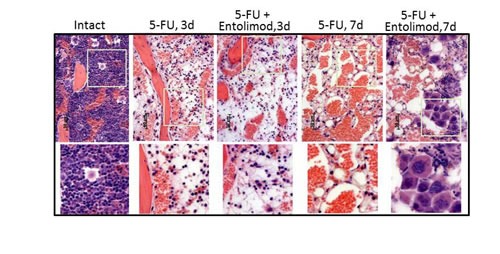
Effect of Entolimod on 5-FU-induced changes in bone marrow morphology Representative pictures of H&E-stained bone marrow sections (10x objective magnification) prepared 3 and 7 days after 5-FU injection. BALB/c mice were treated with 200 mg/kg 5-FU with or without injection of Entolimod 24 and 48 h post-5-FU; 5 mice/group. The lower row shows selected areas outlined in white at two fold higher magnification.

The effect of Entolimod treatment on 5-FU-induced HP damage in BALB/c mice was also assessed through morphological analysis of H&E-stained bone marrow (BM) sections (Fig. 3). This showed that application of 200 mg/kg 5-FU resulted in severe aplasia of the BM, with near-complete absence of HP cells, remnants of stromal cells, expanded sinusoids and hemorrhage observed in BM sections prepared on Day 3 (72 h) after 5-FU administration. Treatment with Entolimod (1 μg/mouse) 24 and 48 h after 200 mg/kg 5-FU had no or very little beneficial effect on BM morphology at this time point. In both 5-FU-treated mice and those given 5-FU in combination with Entolimod, the BM was drastically depopulated in contrast to BM samples from mice treated with Entolimod alone or intact mice which displayed normal morphology with a meshwork of bony spicules, open areas with active hematopoiesis and sinusoids filled with erythrocytes.

At Day 7, the BM of mice treated with 200 mg/kg 5-FU alone contained only single HP and stromal cells that were rounded and detached from each other (indicative of necrosis and necrobiosis). In addition, hemorrhage and an abundant fat component were observed in these BM samples. In contrast, the BM of mice injected with Entolimod after 200 mg/kg 5-FU showed signs of HP recovery including islets of preserved HP cells with prevalent hyperplastic megakaryocytes suggesting that Entolimod promotes recovery of hematopoiesis in the BM and restoration of peripheral HP cell populations after 5-FU-induced damage.

Taken together, these data demonstrate that 5-FU causes severe myelosuppression, neutropenia and thrombocytopenia which likely contribute to the lethality of the treatment in BALB/c mice. Furthermore, Entolimod promotes recovery of hematopoiesis in the BM and restoration of peripheral HP cell populations after 5-FU-induced damage.

### Effect of Entolimod on 5-FU-induced gastrointestinal damage

Previous studies in mice have shown that 5-FU can induce GI damage in the small and large intestines, causing diarrhea and body weight loss leading to mortality [26, 28, 29]. To assess the effect of Entolimod treatment on the GI toxicity of 5-FU, we compared tissue morphology in H&E-stained transverse sections of small and large intestines from untreated “intact” mice and mice treated with different regimens of 5-FU with or without Entolimod. Specific morphological features were evaluated, including (a) the height and width of villi of the small intestines and number of surface enterocytes and goblet cells in the small and large intestines; (b) status of crypts (depth, size and shape, presence of apoptotic bodies, number of and granules in Paneth's cells, luminal migration of epithelial nuclei, loss of goblet cells, presence of atrophy and distortion); and (c) state of the lamina propria (presence of transitory cells, lymphoid accumulations, edema, blood vessel congestion and hemorrhage). The extent of damage related to these features was scored according to the following semi-quantitative scale: 4 – Severe, 3 – Markedly abnormal, 2 – Moderate, 1 – Mild and 0 – No damage.

**Figure 4:**
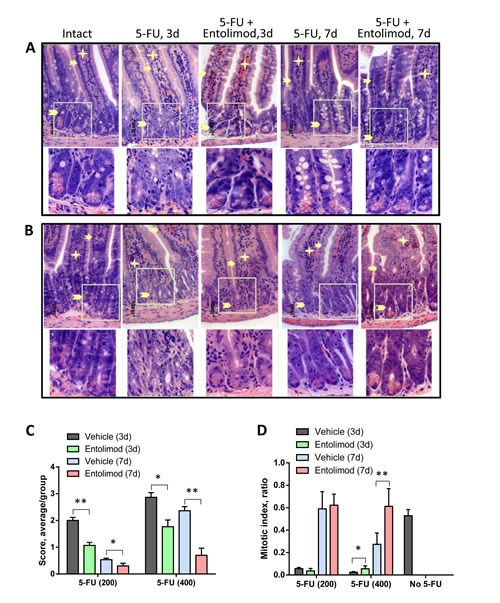
Effect of Entolimod on 5-FU-induced changes in small intestine morphology A-B. Representative H&E-stained transverse sections (250x objective magnification) of small intestines from vehicle injected BALB/c mice (intact) and treated with 5-FU (200 mg/kg (A) or 400 mg/kg (B)) alone or in combination with Entolimod 24 and 48 h post-5-FU. Sections were prepared 3 and 7 days after 5-FU treatment. Enterocytes lining the villi are indicated by arrows, lamina propria by asterisks, and crypts by arrowheads. The lower row shows selected crypt areas outlined in white at two fold higher magnification. C. The average injury score of small intestine sections, as described in (A-B). The degree of pathimorphological changes in the surface epithelium, villi, crypts, lamina propria, stroma, transitory and lymphoid elements and submucosa was scored as: 4 – Severe; 3 – Markedly abnormal; 2 – Moderately abnormal; 1 – Mild; and 0 – Normal, including non-integer scores. The average morphological score for 5 mice/group is shown ± SEM. Differences between corresponding 5-FU and 5-FU+Entolimod groups were statistically significant: (*) p<0.05; (**) p<0.001. D. Mitotic index in mice treated as described in (A-B) was calculated in crypts of 4 transverse sections of small intestine per mouse as the number of mitoses per crypt (12 samples/ group). The mitotic index of vehicle treated animals is plotted as “No-5-FU” control; mean ± SEM; (*) Differences between corresponding 5-FU and 5-FU+Entolimod groups were statistically significant (p<0.05).

Administration of 5-FU (200 mg/kg or 400 mg/kg) into BALB/c mice caused dose-dependent damage in the small and large intestines. The small intestine showed atrophy and necrosis in the crypts and surface epithelium at 3 and 7 days after 5-FU administration (Fig. 4). 5-FU-induced injury was most pronounced in the crypt layer, with smaller and more dense crypts, scarce and degranulated Paneth's cells, abnormal differentiation and maturation of the crypt epithelium, absence of goblet cells, and enlarged and vesicular nuclei. As expected, the observed toxicity was dose- and time-dependent, with greater damage observed after treatment with 400 mg/kg 5-FU versus 200 mg/kg 5-FU and at day 3 versus day 7 indicating GI recovery at the latter time point. However, after 400 mg/kg 5-FU, crypt damage remained significant even at day 7. In addition to the above described changes in the crypts, severe 5-FU-induced injury was seen in the surface epithelium of the villi, in the lamina propria and in the submucosa. For both tested 5-FU doses, Entolimod injection at 24 and 48 h post-5-FU led to significant mitigation of 5-FU-induced damage to the small intestine mucosa and morphology closer to normal (Fig. 4A-C). The most dramatic evidence of this was the preservation of secretory Paneth's cells in the crypts of all mice treated with Entolimod in addition to 5-FU. The beneficial effect of Entolimod was clearly illustrated by the near-complete restoration of normal small intestine morphology on day 7 after 5-FU injection in Entolimod-treated mice, while those given 5-FU alone showed severe damage with no signs of recovery at the same time point. In addition, the average overall GI injury scores obtained by semi-quantitative scoring of the stained tissue sections confirmed efficacy of Entolimod in reducing small intestine toxicity of 200 or 400 mg/kg doses of 5-FU at both day 3 and day 7 (Fig. 4C).

**Figure 5:**
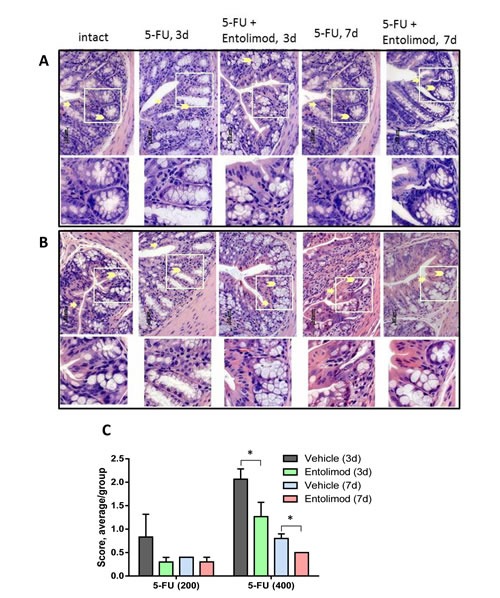
Effect of Entolimod on 5-FU-induced changes in colon morphology A-B. Representative H&E-stained transverse sections (250x objective magnification) of colon from untreated “intact” BALB/c mice and those treated with 5-FU injections (200 mg/kg (A) or 400 mg/kg (B)) alone or in combination with Entolimod treatment 24 and 48 h post-5-FU. Sections were prepared 3 or 7 days after 5-FU treatment. Morphology of crypt areas outlined in white is shown at two fold higher magnification in the lower rows. Atrophic and degenerative changes in the surface epithelium are indicated by arrows and in the crypt cells and mucin globules by arrowheads. C. The average injury score was determined by pathomorphological changes in the surface epithelium, number of surface enterocytes and goblet cells, size and shape of crypts, lymphoid elements and state of the submucosa in colon sections from mice described in (A-B); 5 mice/group; mean ± SEM. Differences between corresponding 5-FU and 5-FU+Entolimod groups were statistically significant (*) p<0.05.

Similar analysis of the morphology of the large intestine (colon) in BALB/c mice after 200 mg/kg or 400 mg/kg 5-FU treatment showed dose-dependent injury of the colonic mucosa on day 3 and 7 post-5-FU, with atrophic and degenerative changes observed mainly in the crypt cells (e.g., loss of mucin globules, loss of distinct boundaries between adjacent cells, disappearance of the apical cytoplasm, and extended crypt lumens) (Fig. 5). Treatment of 5-FU-injected mice with Entolimod led to improved preservation of crypt cells and mucin globules and overall closer-to-normal morphology in the large intestine. The beneficial effect of Entolimod on 5-FU-induced large intestine injury at day 3 and day 7 post-5-FU was found to be statistically significant for groups treated with high dose (400 mg/kg) 5-FU (Fig. 5C). The GI toxicity of 5-FU and potential mitigative effect of Entolimod were examined further by determining the number of mitoses per crypt (“mitotic index”) in transverse small intestine sections as an indicator of the proliferative capacity/health of the crypts (Fig. 4D). 5-FU administration (200 or 400 mg/kg) was found to cause a dose-dependent reduction in mitotic index on day 3 post-injection. At both 5-FU dose levels, Entolimod treatment ameliorated the drop in mitotic index on day 3 (although only significantly so with 400 mg/kg 5-FU). By day 7 after 200 mg/kg 5-FU injection, the mitotic index was restored to normal in both mice treated with 5-FU alone and those treated with 5-FU+Entolimod; however, on day 7 after 400 mg/kg 5-FU injection, efficacy of Entolimod in promoting restoration of the small intestine mitotic index was evident. The mitotic index on day 7 in mice treated with Entolimod after 400 mg/kg 5-FU was the same as in intact mice, while that in mice treated with 5-FU alone remained ~50% lower than normal (Fig. 4D).

Taken together, the results of these direct analyses of the GI tract demonstrate that Entolimod counteracts the GI toxicity of 5-FU, primarily by stimulating regeneration of damaged GI tissue.

### Role of IL-6 in the protective effects of Entolimod against 5-FU toxicity

**Figure 6:**
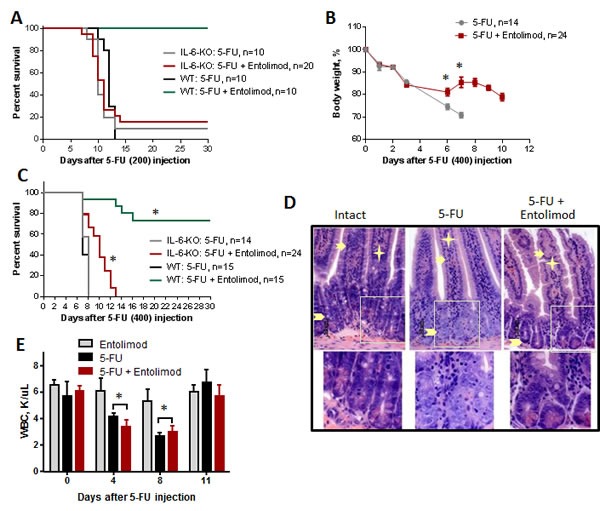
Effect of Entolimod on 5-FU toxicity in IL-6 knockout mice A. Survival of WT BALB/c and IL-6-KO mice after treatment with 200 mg/kg 5-FU, with or without Entolimod treatment (1 μg/mouse) 24 and 48 h after 5-FU. B. The kinetics of body weight changes in IL-6-KO mice treated with 5-FU (400 mg/kg) and Entolimod injected 24 and 48 h after the last 5-FU dose (mean ± SEM). C. Survival of IL-6-KO and wild type (WT) mice treated with 5-FU (400 mg/kg) or 5-FU+Entolimod. The combined results of two independent experiments are presented in (B) and (C). (*) Differences between corresponding 5-FU and 5-FU+Entolimod groups were statistically significant (p<0.001). D. H&E-stained small intestine sections showing crypts from IL-6-KO mice euthanized on Day 3 after 5-FU (400 mg/kg) with or without Entolimod. Enterocytes lining the villi are indicated by arrows, lamina propria by asterisks and crypts by arrowheads. Normal morphology of crypts in an intact mouse is shown as a control. E. Concentrations of white blood cells (WBC) in blood samples from IL-6-KO mice treated with Entolimod alone (n=3), 5-FU alone (n=5) or 5-FU+Entolimod (n=5). 5-FU (100 mg/kg) was followed by Entolimod 24 and 48 h later; m ± SEM. (**) Differences between the 5-FU and 5-FU+Entolimod groups were not statistically significant (p<0.05).

The NF-B-regulated cytokine IL-6 is strongly upregulated in response to Entolimod treatment [30]. IL-6 is a candidate mediator of the tissue protective effects of Entolimod since it is known to have protective and hematopoiesis stimulating properties (particularly for the thrombocytic lineage) [30-33]. Induction of IL-6 by Entolimod was previously shown to be critical for mitigation of HP acute radiation syndrome and prevention of death in mice after total body irradiation [30]. Here, we found that Entolimod injection also causes induction of IL-6 in 5-FU-treated mice. A strong Entolimod-dependent spike in plasma levels of IL-6 (58-fold over the level observed with vehicle treatment) was observed in BALB/c mice at 7 days after 200 mg/kg 3-FU treatment (Supplementary Fig. S4). In order to determine whether Entolimod-induced IL-6 plays a role in the drug's ability to protect mice against 5-FU toxicity, we compared the effects of Entolimod treatment on 5-FU-associated body weight loss, mortality and morphological GI damage in IL-6-KO mice versus wild type (WT) BALB/c mice. IL-6-KO and WT mice displayed similar sensitivity to 3 different does of 5-FU as determined by body weight loss and survival (100, 150 and 200 mg/kg) (data not shown). However, while Entolimod was able to rescue WT mice from death induced by 200 mg/kg 5-FU, it did not reduce the lethality of the same dose of 5-FU in IL-6-KO mice (Fig. 6A). In contrast, with a higher dose of 5-FU (400 mg/kg), Entolimod treatment did provide some survival benefit to IL-6-KO mice, increasing mean survival time from 8 to 10 days (P<0.001) (Fig. 6C). This improvement in survival of IL-6-KO mice dosed with 400 mg/kg 5-FU was associated with Entolimod-mediated recovery of body weight after initial 5-FU-induced weight loss (Fig. 6B), consistent with mitigation of GI damage in KO animals with preservation of crypt cells in small intestine and higher mitotic index (Fig. 6D and Supplementary Fig. S5) as in WT animals (Fig. 4B and D). Entolimod did not, however, prevent mouse lethality and all KO mice treated with 400 mg/kg 5-FU + Entolimod died by day 13 post-5-FU (Fig. 6C). Using a sub-lethal dose of 5-FU (100 mg/kg), we confirmed that Entolimod-mediated acceleration of HP recovery was absent in IL-6-KO mice (Fig. 6E) in contrast to WT mice (Fig. 2B). Together with the knowledge that in BALB/c mice 200 and 400 mg/kg doses of 5-FU induce primarily HP and GI damage, respectively, these data demonstrate that IL-6 production is an essential mechanism of Entolimod-mediated restoration of hematopoiesis after 5-FU treatment, but is not required for mitigation of GI toxicity under the same conditions.

In addition to its direct cytotoxic effects, 5-FU toxicity can be mediated by the development of inflammation following from induction of pro-inflammatory cytokines and ROS formation in GI tissues. In particular, IL-1 has been identified as an essential mediator of 5-FU GI toxicity [26]. Natural mechanisms by which IL-1 activity can be suppressed include increased production of soluble IL-1 receptor and/or IL-1 receptor antagonist (IL-1RN) proteins [34]. Notably, IL-1RN expression was previously shown to be induced in the colon of flagellin-treated mice and to contribute to the amelioration of colitis in the treated mice [14]. Here, we found that the plasma concentration of soluble IL-1 receptor was significantly elevated in mice treated with Entolimod alone or following 5-FU as compared to untreated “intact” mice (Supplementary Fig. S6A). In addition, upregulation of IL-1RN expression in the small intestine was detected following Entolimod treatment of mice (Supplementary Fig. S6B). These data suggest neutralization of IL-1 activity as a possible mechanism underlying Entolimod-mediated mitigation of 5-FU toxicity in GI tissues. Additionally, our finding that production of the antioxidant enzyme SOD2 in the small intestine was increased following Entolimod treatment of mice presents neutralization of ROS as another possible mechanism involved in the protective effects of Entolimod against 5-FU-induced tissue damage (Supplementary Fig. S6C, [35]).

### Entolimod does not protect tumors from 5-FU

**Figure 7:**
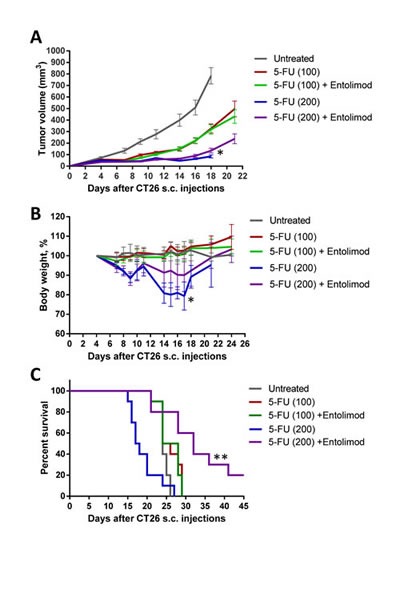
Protection of mice, but not tumors, from 5-FU toxicity by Entolimod in CT26 tumor-bearing mice A. Kinetics of s.c. CT26 tumor growth in mice treated with vehicle (“untreated”), 5-FU alone (100 or 200 mg/kg), or 5-FU + Entolimod injected 1, 48, and 96 h post-5-FU (10 mice, 20 tumors per group in 5-FU treated groups, and 5 mice, 10 tumors, in “untreated” group, mean ± SEM). B. The kinetics of body weight changes (percentage of starting weight) and (C) survival of mice described in A. (*) – Body weight and tumor volume are shown for only 4/10 mice injected with 200 mg/kg 5-FU alone that were surviving on Day 18 after CT26 cell inoculation; mice in this group died due to 5-FU toxicity with small or no tumors. In the other groups, mice were euthanized due to large size or ulceration of tumors; 2/10 mice in the group treated with 200 mg/kg 5-FU + Entolimod remained tumor-free for the entire period of observation (60 days). (**) Differences in mouse survival between 5-FU (200 mg/kg) + Entolimod group and any other group were statistically significant (p<0.05 by Log-rank test for mean survival).

Clearly, use of Entolimod to improve anticancer chemotherapy by reducing its adverse side effects requires that the protective effects of the drug are strictly limited to normal (non-tumor) cells and tissues. Since 5-FU is commonly used as a single agent or in combination with other chemotherapeutic drugs to treat colorectal cancer [36, 37], we used the mouse CT26 colorectal carcinoma model to test the effect of Entolimod on tumor sensitivity to 5-FU. CT26 cells do not express TLR5 and their growth as s.c. tumors is not suppressed by Entolimod treatment (Supplementary Fig. S7) [10]. CT26 tumors were, however, sensitive to 5-FU treatment as expected, showing 5-FU dose-dependent reduction of tumor growth (Fig. 7A). Inclusion of Entolimod in the treatment regimen with either 100 mg/kg or 200 mg/kg 5-FU did not have any significant effect on 5-FU-induced tumor suppression (Fig. 7A).

Entolimod treatment also did not significantly change survival of tumor-bearing mice treated with 100 mg/kg 5-FU; 100% of mice in these two groups were euthanized by Day 29 after tumor cell inoculation due to tumors reaching the size endpoint or developing ulcerations (Fig. 7B). In contrast, 100 % of mice injected with 200 mg/kg 5-FU without Entolimod died due to 5-FU toxicity by Day 27 post tumor cell implantation (Day 23 post 5-FU injection), while those treated with the combination of 200 mg/kg 5-FU and Entolimod experienced significantly less toxicity. Tumor-bearing mice given Entolimod after 200 mg/kg 5-FU displayed less weight loss (Fig. 7B) and significantly prolonged survival (Fig. 7C) compared to those treated with 200 mg/kg 5-FU alone. Moreover, 2 of the 10 mice treated with 200 mg/kg 5-FU+Entolimod survived tumor-free for the entire period of observation (>60 days). These results illustrate that treatment of CT26 tumor-bearing mice with Entolimod in combination with 5-FU reduced the systemic toxicity of 5-FU without affecting its antitumor efficacy.

## DISCUSSION

Our previous work showing that the TLR5 agonist Entolimod rescues mice and non-human primates from lethal irradiation by protecting and stimulating regeneration of HP and GI tissues [7] suggested that the drug might have similar protective effects in the context of genotoxic chemotherapy. Systemic administration of Entolimod leads to NF-B- and STAT3-mediated induction of numerous bioactive factors including anti-apoptotic proteins, scavengers of ROS, cytokines and anti-inflammatory agents which can contribute to protection of normal tissues from the damaging effects of radiation- or drug-related genotoxicity [10, 38]. For example, one of the strongest responders to Entolimod treatment IL-6 is known to have protective properties through activation of the STAT3 signaling [31, 39-42].

Here, using 5-FU as a model, we show that Entolimod is indeed capable of reducing the toxicity of genotoxic chemotherapy to GI and HP tissues resulting in overall improvement in health and survival of treated mice. Entolimod did not prevent 5-FU-induced depletion of HP cell populations, but did accelerate their recovery to normal levels in an IL-6-dependent manner. This suggests that Entolimod does not directly interfere with 5-FU HP toxicity, but rather reduces the severity of damage and stimulates tissue regeneration. In the GI tract, Entolimod treatment both reduced the extent of 5-FU-induced damage and promoted tissue recovery, but Entolimod-stimulated IL-6 production was not responsible for these effects. Elevated levels of the antioxidant enzyme SOD2 and IL-1 receptor antagonist (IL-1RN) detected in the small intestine and inhibitory soluble IL-1 receptor detected in the plasma of Entolimod-treated mice suggest that reduction of ROS activity and inhibition of IL-1-mediated inflammation may contribute to the Entolimod's ability to reduce the GI toxicity of 5-FU [26]. The latter possibility is consistent with previous work showing that induction of IL-1RN in intestinal epithelia and macrophages following flagellin-mediated TLR5 stimulation was important for limiting inflammatory responses to Salmonella infection and reducing inflammasome-associated colitis [14, 43].

A major potential concern with applying Entolimod to reduce the toxicity of chemotherapeutic drugs is that the protective/ regenerative effects of the drug could potentially reduce the antitumor efficacy of the chemotherapy. However, in numerous experiments with multiple mouse tumor models (e.g., colon adenocarcinoma CT26, breast 4T1, etc.), we have never observed any stimulation of tumor growth or induction of tumor recurrence by Entolimod treatment [7, 8, 10]. In the current study, mice bearing syngeneic CT26 tumors were protected from the systemic toxicity associated with 5-FU treatment by injection of Entolimod, but Entolimod did not reduce the tumor suppressive effect of 5-FU. The difference between normal and tumor cell responses to Entolimod is likely due to the fact that most tumor cells commonly acquire impairment of pathways controlling cell cycle and DNA repair (e.g., p53), frequently resulting in constitutively active NF-B and STAT3 [38, 44, 45] thus making TLR5 stimulation ineffective. In addition to its failure to protect tumors from radiation and chemotherapy, Entolimod actually demonstrates antitumor efficacy in itself. This has been shown for subcutaneously growing TLR5-positive tumors as well as tumors growing in TLR5-positive tissues (e.g., liver) regardless of the TLR5 status of the tumor cells [8, 10]. The tumor suppressive effects of Entolimod appear to be due to induction of cytokines leading to development of antitumor immune responses [11, 12]. Therefore, we have multiple reasons to expect that Entolimod has strong potential to improve the therapeutic index of anticancer chemotherapeutics by mitigation of chemotherapy-induced adverse side effects in hematopoietic and gastrointestinal tissues without reducing tumor suppression.

## MATERIALS AND METHODS

### Mice

BALB/c and C57BL/6 female mice, 10-14 weeks old (Jackson Laboratory, Bar Harbor, ME) were used in the study. IL-6 deficient mice C.129S2-IL6^tm1/kopf^/J (BALB/c background) were originally purchased from Jackson Laboratory; TLR5 knockout mice B6.129P2-Tlr5^tm1Aki^ (C57BL/6 background) were a generous gift of Dr. Shizuo Akira (University of Tokyo, Japan); mice of both strains were bred at Roswell Park Cancer Institute (RPCI). All animal experiments followed protocols approved by the RPCI IACUC.

### Reagents

Entolimod™, a TLR5 agonistic agent [7] was obtained from Cleveland BioLabs, Inc. (Buffalo, NY). 5-Fluorouracil (5-FU) was purchased from Sigma (St. Louis, MO).

### Tumor cells

Murine colon undifferentiated carcinoma CT26 cells (ATCC) were cultured in RPMI media with 10% FBS, standard supplements (2 mmol/L L-glutamine, 100mol/L nonessential amino acids) and 1% penicillin-streptomycin (Invitrogen) at 37°C in a 5% CO_2_ incubator.

### *In vivo* model of 5-FU toxicity

Mice were weighed and randomly divided into treatment groups. 5-FU was diluted in DMSO (40%) and injected i.p. (100 or 200 mg/kg / 200 μl/ injection). High dose 5-FU (400 mg/kg) was given as two i.p. injections of 200 mg/kg 6 h apart. Entolimod (1 μg/ 100 μl) was injected s.c. at the indicated times after the first 5-FU injection. When mice were given three i.p. injections of 5-FU (100 mg/kg/day, once/day), Entolimod (1 μg/mouse)was injected 24 and 48 h after the last 5-FU injection. DMSO (40%) and PBS were used as vehicle controls for 5-FU and Entolimod, respectively. Mice were monitored daily or at least 3 times a week for survival and signs of morbidity, including changes in body weight.

### *In vivo* model of 5-FU antitumor therapy

CT26 tumor cells (5x10^5^ /100 μl PBS) were injected s.c. into syngeneic BALB/c mice (2 flanks/mouse). When tumors reached ~5 mm in diameter, mice were randomly divided into 5 treatment groups. 5-FU (100 or 200 mg/kg) was injected i.p. with Entolimod (1 μg/ 100 μl) or PBS injected s.c. 1, 48 and 96 h after 5-FU. A group of mice injected with vehicle solutions was used as an “untreated” control. Tumors were measured by two diameters with a digital caliper every second day. Tumor volume (V) was calculated as: V= x d_1_^2^ x d_2_, where d_1_< d_2_. Mice were euthanized according to IACUC protocol when signs of morbidity were observed or tumors reached about 13 mm in diameter or developed ulceration.

### Complete blood cell analysis

25 μL of whole blood from the orbital sinus were analyzed to determine complete and differential blood cell counts using a Hemavet 950 Hematology System (Drew Scientific, Dallas, TX).

### Histological analysis

Immediately after euthanasia on day 3 or 7 after 5-FU injection, 1.0 cm samples of duodenum, jejunum, ileum, colon and femora with bone marrow (BM) were harvested from mice processed for hematoxylin-eosin (H&E) staining and analyzed in a blinded fashion by a trained pathologist using ImagePro software (at 10x magnification unless otherwise stated). Pathomorphological changes in intestinal sections were scored as: 4 – Severe; 3 – Markedly abnormal; 2 – Moderate; 1 – Mild l; or 0 – Normal, with intermediate non-integer scores assigned based on the pathologist's judgment. The mitotic index was determined by counting the mitotic figures in crypt cells of the small intestine using light microscopy at 100× magnification. The mitotic index is calculated in longitudinally sectioned crypts as an average value per crypt in each treatment-group (4 samples per mouse, 3 mice/group).

### Statistical analysis

Differences in body weight loss (expressed as percent initial body weight) between groups were analyzed by two-way (time and treatment) repeated measures ANOVA; differences in histomorphological scores of tissue damage and mitotic index were analyzed by Student's t-test (two-tailed, unequal variances); differences in survival kinetics (mean survival time) were analyzed by log-rank test using GraphPad Prism software; differences in discrete blood cell populations between treatment groups were determined using two-tailed unpaired Student's t-test. Significance level was set at P <0.05.

## SUPPLEMENTARY FIGURES AND METHODS




