# Supplementary material for: Toll-like receptor-5 agonist Entolimod broadens the therapeutic window of 5-fluorouracil by reducing its toxicity to normal tissues in mice
Source: Oncotarget. 2014 Feb 23;5(3):802–14. doi: 10.18632/oncotarget.1773 (PMC3996654; doi:10.18632/oncotarget.1773)
Supplement: Supplementary file 1 [file oncotarget-05-802-s001.pdf]

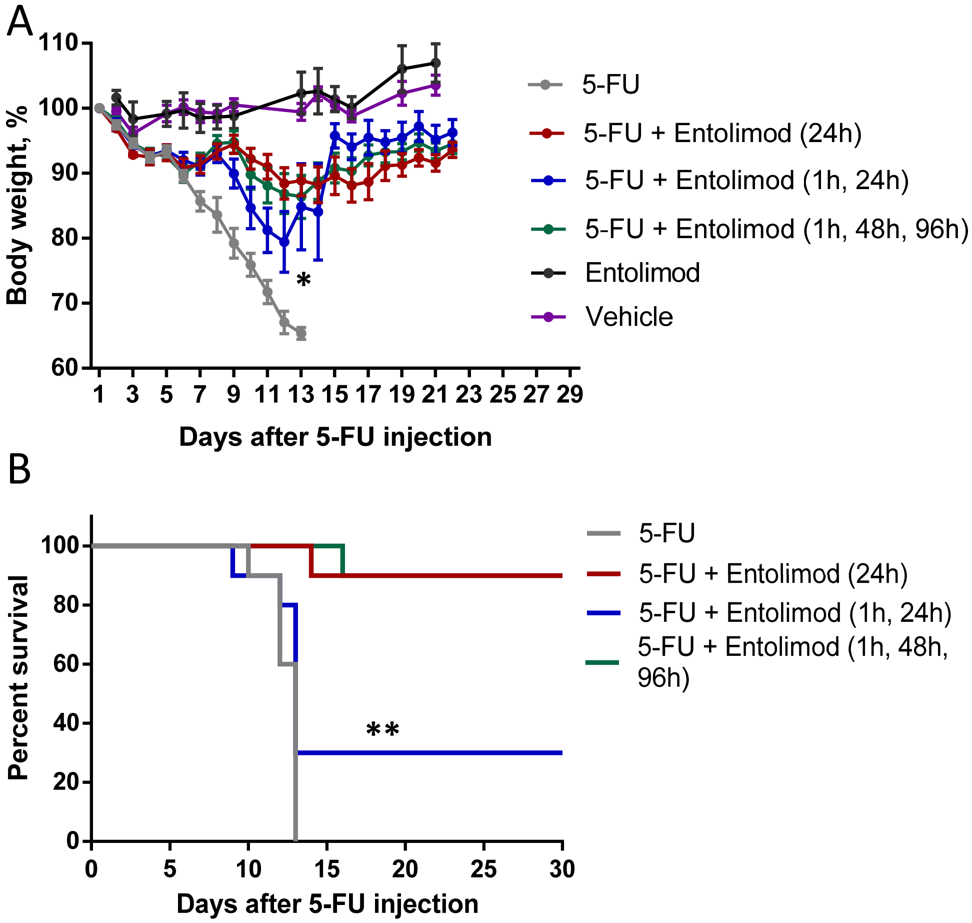

Supplementary Fig. S1. Mitigation of body weight loss and increase of survival of BALB/c mice after 5-FU injections (200 mg/kg) by Entolimod treatment. (A) Body weight and (B) survival of BALB/c mice after 5-FU (200 mg/kg) with and without Entolimod injections given in different regimens: 24 h post-5-FU, 1 and 24 h post-5-FU, and 1, 48 and 96h post-5-FU; n=10 mice/group. Entolimod and vehicle injected mice without 5-FU were weighed as controls. Mean body weight (as a percentage of starting weight) +/- SEM is shown. (*) Body weight beyond this time-point is based on 3 surviving mice in the group treated with Entolimod 1 and 24 h post-5-FU. (**) Not statistically significant difference from 5-FU alone (p>0.05).

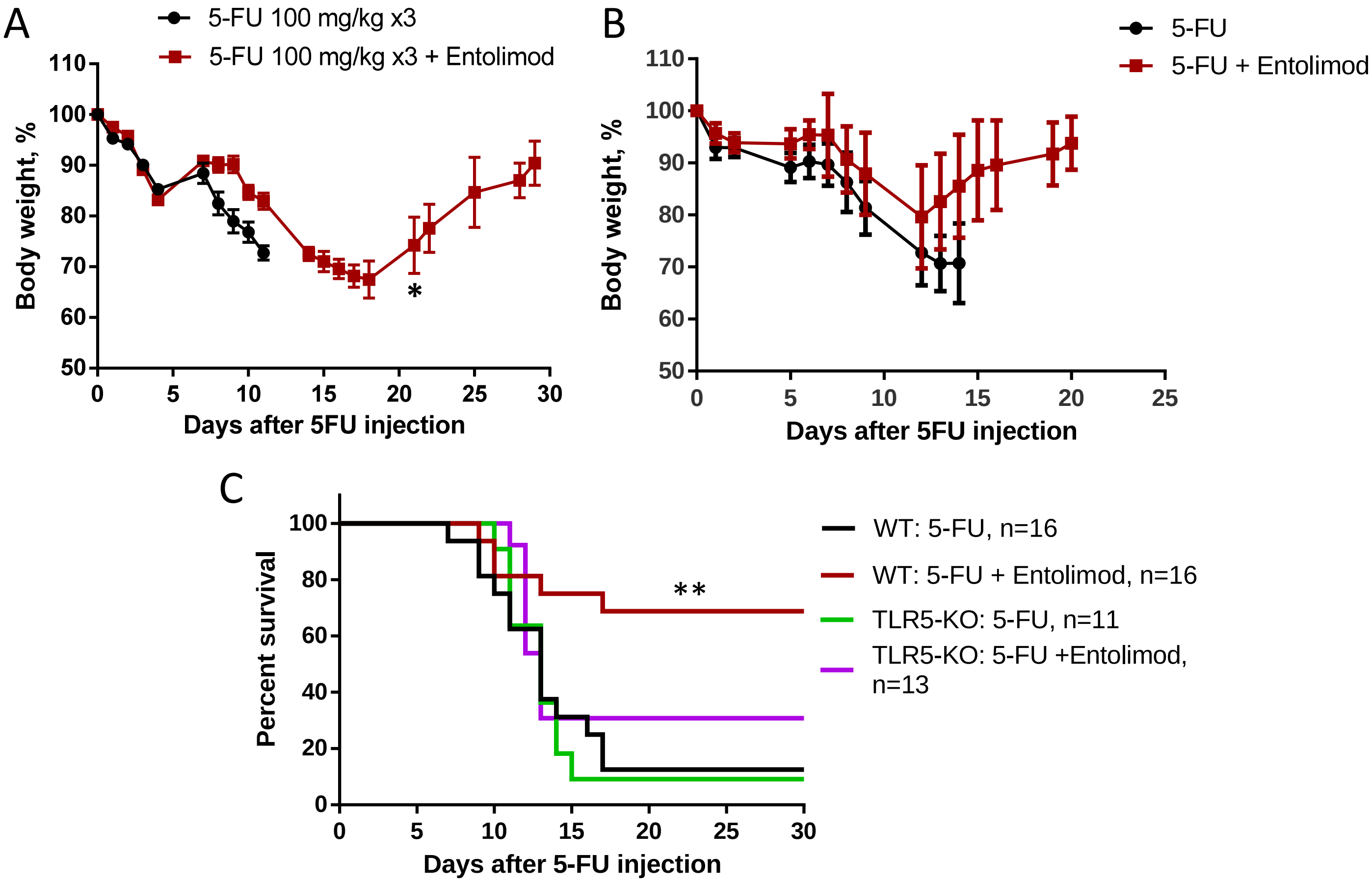

Supplementary Fig. S2. Entolimod-mediated protection against 5-FU toxicity is TLR5-dependent. Body weight changes in wild type C57BL/c mice injected with 5-FU: (A) 100 mg/kg x 3 times with 24 h interval with Entolimod injected 24 and 48 h after the last 5-FU treatment, and (B) 200 mg/kg x 2 times with 6 h interval with and without Entolimod injected 24 and 48 h post-5-FU (B); n=10; (*) The results of the group treated with 5-FU and Entolimod at this point and later are based on 3 surviving mice. C. Survival of wild type C57BL/6 and TLR5-KO mice injected with 5-FU – 200 mg/kg x 2 times with 6 h interval with and without Entolimod injected 24 and 48 h post-5-FU. (**) p=0.0022 for comparison of WT mice treated with 5-FU+Entolimod to WT mice treated with 5-FU only.

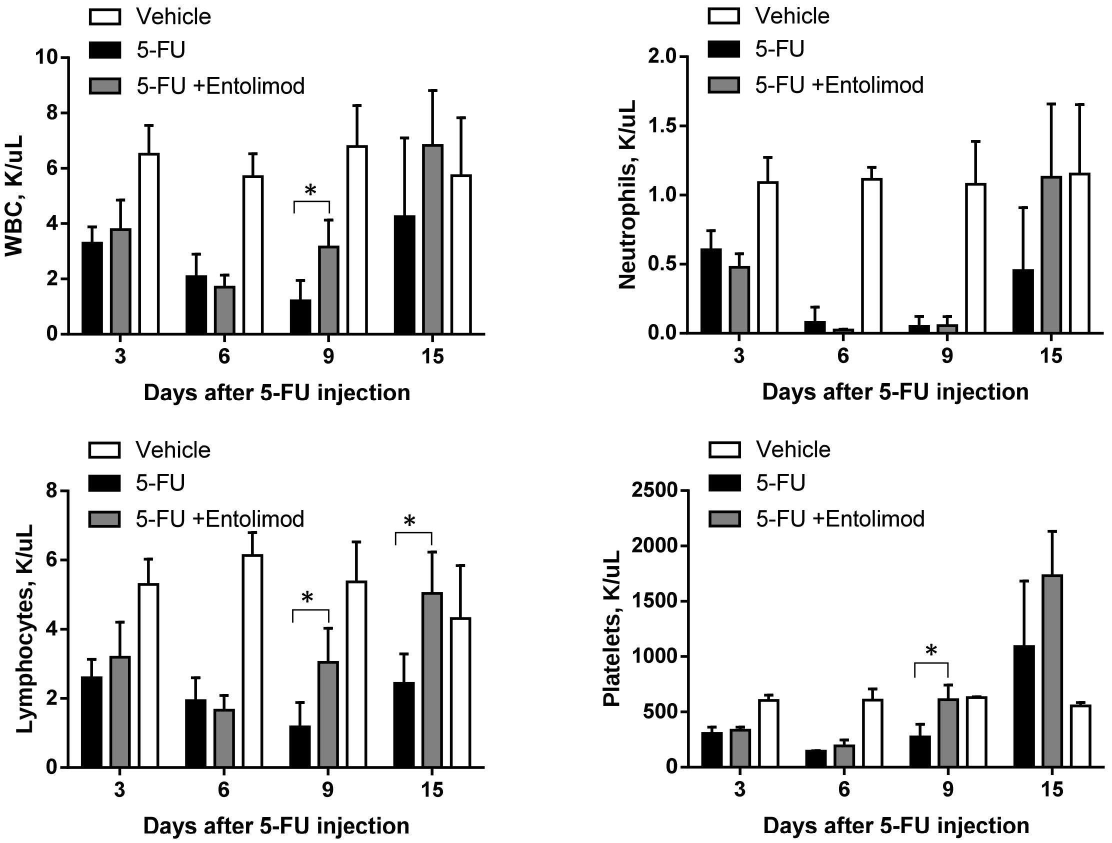

Supplementary Fig. S3. Restoration of blood cell count in BALB/c mice following 5-FU treatment alone and in combination with Entolimod. 5-FU was injected i.p. (150 mg/kg) followed by Entolimod 24 h and 48 h post 5-FU (n=5 mice/group). Two out of 5 mice in the 5-FU injected group died on day 8, all 5 mice in the 5-FU+Entolimod-treated group survived. Vehicle injected mice were used as controls (n=3-5). (*) The difference is statistically significant ($p<0.05$).

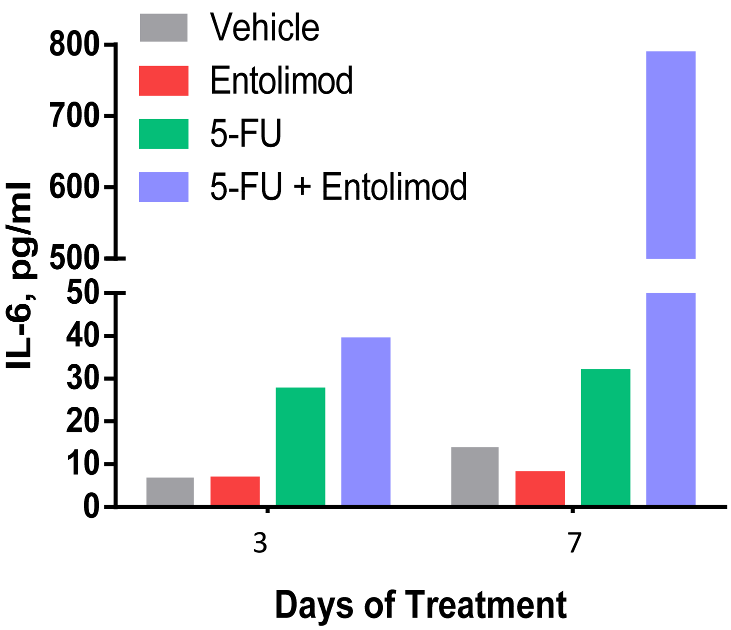

Supplementary Fig. S4. Entolimod induces IL-6 production in 5-FU-treated mice. IL-6 concentration in plasma samples from BALB/c mice was analyzed by MILLIPLEX kit (average of 2 pools of 3 mouse samples each at each data-point). Plasma was collected 3 and 7 days after injection of vehicle (DMSO), Entolimod alone, 200 mg/kg 5-FU alone, or 5-FU+Entolimod.

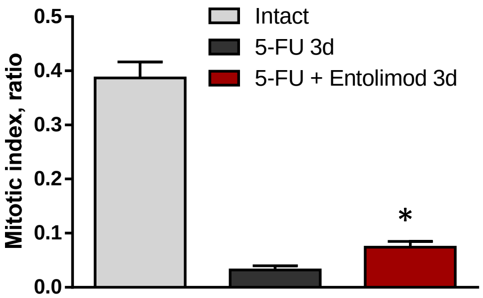

Supplementary Fig. S5. Mitotic index in IL-6-KO mice after 5-FUtreatment with and without Entolimod. Mitotic index was calculated in crypts of 4 transverse sections of small intestine per mouse as the number of mitoses per crypt in 3 mice/ group. The samples were obtained 3 days after 5-FU (400 mg/kg) injections with and without Entolimod 24 and 48 h post-5-FU (12 samples/ group). Mean ± SEM is shown. (*) The difference in mitotic index between 5-FU and 5-FU+Entolimod groups was statistically significant ($p<0.05$).

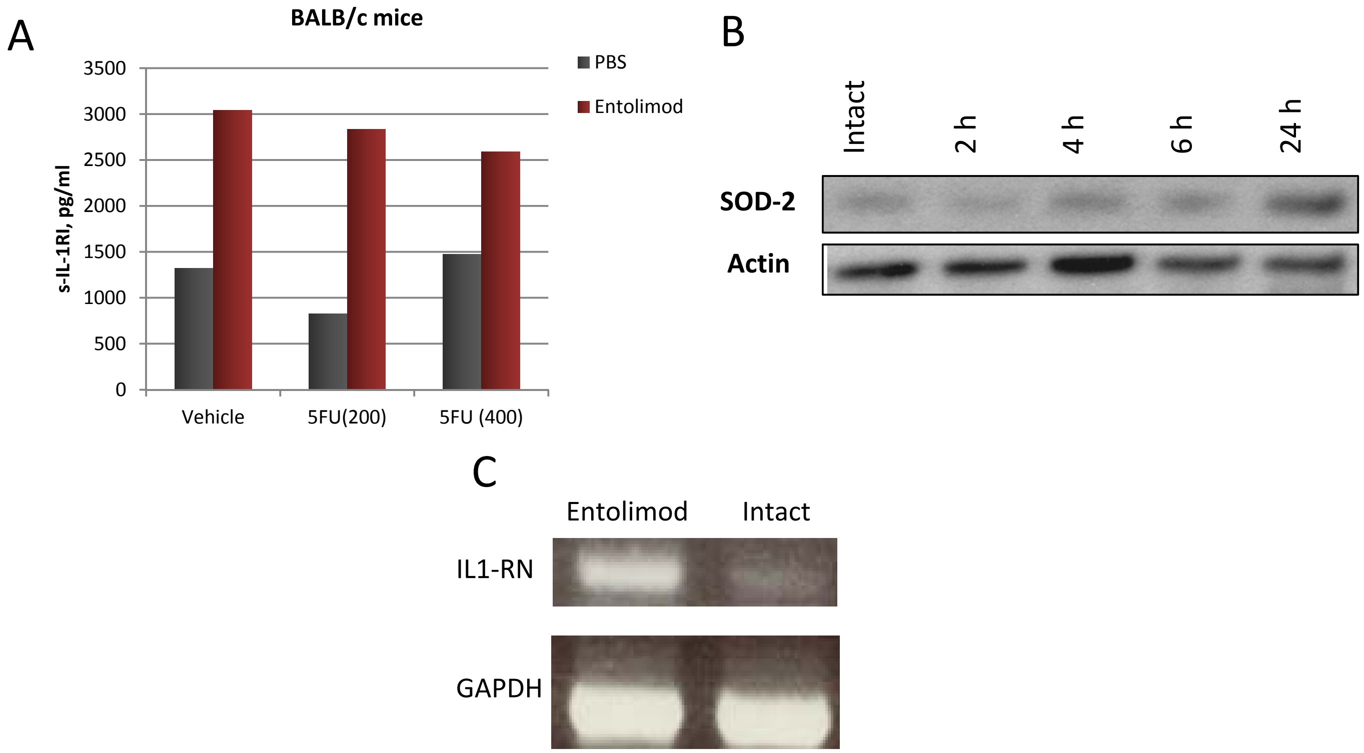

Supplementary Fig. S6. Entolimod induces expression of soluble IL-1 receptor, SOD2, and IL-1 receptor antagonist (IL1-RN). (A) The concentration of soluble IL-1 receptor in plasma prepared from BALB/c mice 72 h after 5-FU injections (200 and 400 mg/kg) with and without Entolimod injected 24 and 48 h after 5-FU was determined using MILLIPLEX kit (average of 2 pools of 3 mouse samples at each data-point). (B) Western blotting for SOD-2 expression in small intestine samples from NIH-Swiss mice at the indicated time-points after Entolimod injection (without 5-FU). Actin was used as a loading control. (C) Expression of IL-1 receptor antagonist (IL-1RN) was detected by RT-PCR using total RNA prepared from small intestine isolated from a BALB/c mouse treated with Entolimod (1 μg/ mouse x2 injections 24h apart) 24 h after the second injection. Small intestine RNA from a PBS-injected mouse was used as an "intact" control. GADPH expression was used a housekeeping gene for loading control. Primers: IL1-RN (220 bp product): Forward: 5'-TAG CAA ATG AGC CAC AGA CG-3', Reverse: 5'-ACA TGG CAA ACA ACA CAG GA-3' and GAPDH (400-500 bp product): Forward: 5'-ACC ACA GTC CAT GCC ATC AC-3', Reverse: 5'-TCC ACC ATG TTG CTG TA-3'.

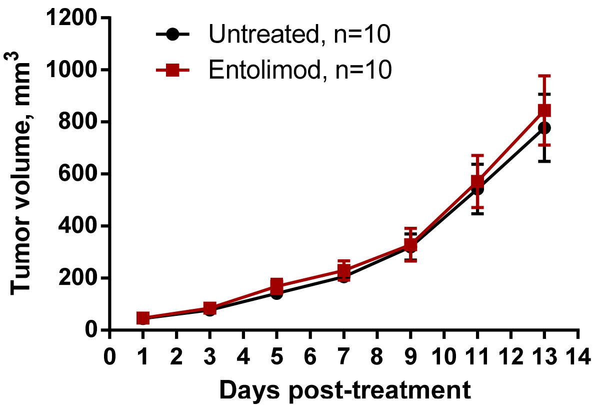

Supplementary Fig. S7. Representative experiment showing the absence of any effect of Entolimod on s.c. growing CT26 tumors in syngeneic BALB/c mice. Treatment of CT-26 tumor-bearing mice with Entolimod was initiated when tumors reached about 5 mm in diameter. Entolimod (1 μg/mouse) was injected s.c. 24 h apart on days 1, 2 and 3; mean ±SEM, 5 mice x2 tumors per group (n=10).
