# Supplementary material for: Toll-like receptor-5 agonist Entolimod broadens the therapeutic window of 5-fluorouracil by reducing its toxicity to normal tissues in mice
Source: Oncotarget. 2014 Feb 23;5(3):802–14. doi: 10.18632/oncotarget.1773 (PMC3996654; doi:10.18632/oncotarget.1773)
Supplement: Supplementary file 2 [file oncotarget-05-802-s002.pdf]

Supplementary Methods

Cytokine analysis

Concentrations of IL-6 and soluble IL-1 receptor in mouse plasma were determined using MILLIPLEX kit (EMD Millipore, St. Charles, MO). Plasma was prepared from blood collected from BALB/c mice by heart puncture upon euthanasia 3 or 7 days after 5-FU injections (200 mg/kg) with or without Entolimod (1 μg/ mouse) injected 24 and 48 h after 5-FU. Plasma from vehicle-injected mice was used as an untreated control. There were 5 mice per treatment group and plasma from 2-3 mice was pooled together for analysis (data is shown as the average of 2 pools for each treatment group).

Western blotting

The levels of SOD-2 expression in mouse small intestine samples collected 2, 4, 6 or 24 h after Entolimod (1 μg/ mouse) s.c. injections were determined by Western blot analysis using rabbit polyclonal anti-SOD-2 antibody followed by secondary goat anti-rabbit HRP-conjugated antibody (Santa Cruz Biotechnology, Inc.). The small intestine of a PBS-injected mouse was used as an "intact" control. β-actin was analyzed as a loading control.

RT-PCR

Expression of IL-1 receptor antagonist (IL-1RN) was detected by RT-PCR using total RNA prepared from the small intestine isolated from a mouse treated with Entolimod (1 μg/ mouse x2 injections 24 h apart) 24 h after the second injection. The small intestine from a PBS-injected mouse was used as an "intact" control. Total RNA was extracted using TRIzol reagent according to manufacturer's instructions (Invitrogen, Carlsbad, CA). cDNAs were synthesized using iScript™ cDNA Synthesis Kit (Bio-Rad Laboratories, Hercules, CA) according to manufacturer's protocol from 250 ng RNA/sample. 1 μL cDNA in 12.5 μL TAQ PCR Master

Mix, 2X (Affymetrix, Santa Clara, CA) and 9.5 μL DNase, RNase free water was amplified by PCR using forward and reverseprimers for IL-1RN and GAPDH (house-keeping gene for loading control): IL-1RN (220 bp product): Forward: 5'-TAG CAA ATG AGC CAC AGA CG-3', Reverse: 5'-ACA TGG CAA ACA ACA CAG GA-3' and GAPDH (400-500 bp product): Forward: 5'-ACC ACA GTC CAT GCC ATC AC-3', Reverse: 5'TCC ACC ATG TTG CTG TA-3'. The RT-PCR conditions were 95°C for 2 min, followed by 32 cycles for IL-1RN and 25 cycles for GAPDH of 95°C for 30 sec, 55.2°C (IL-1RN) or 52.1°C (GAPDH) for 30 sec, and 72°C for 1 min, and 1 cycle of 72°C for 3 min (for final extension). The PCR products were visualized on 1.2% agarose gels stained with ethidium bromide.
